# Effects of Temperature Dependence in Mosquito Mortality on Simulated Chikungunya Virus Transmission

**DOI:** 10.3390/v17111486

**Published:** 2025-11-08

**Authors:** Cynthia C. Lord

**Affiliations:** Florida Medical Entomology Lab, University of Florida-IFAS, Vero Beach, FL 32962, USA; clord@ufl.edu

**Keywords:** Chikungunya virus, *Aedes albopictus*, *Aedes aegypti*, mosquito mortality, model

## Abstract

A compartmental, deterministic model was used to explore the effects of temperature dependency in mosquito mortality on the likelihood of epidemics and the size of outbreaks of Chikungunya virus under Florida temperature conditions. Two known vectors, *Aedes albopictus* and *Ae. aegypti*, were included, with similar structure but allowing mortality and abundance parameters to vary between them. The mortality relationship with temperature had a central optimal survival region, with increasing mortality outside these regions. The central temperature and the annual mean temperature were most influential in the likelihood of an epidemic, although the variance explained was low. The central temperature, annual mean temperature and day of virus infection influenced the size of the outbreaks. Regression models including two-way interactions explained more of the variance in outcomes than the main effects models, but there was still substantial variance left unexplained. Given the model structure, higher order interactions would be required to explain most of the variance.

## 1. Introduction

Vector mortality has been identified as a critical component of vector-borne pathogen transmission in many models (e.g., [1,2,3,4]; reviews in [5,6,7]). Increased survival (lower mortality) allows more individuals to survive the extrinsic incubation period and have a higher expectation of infectious life, increasing vectorial capacity and R_0_. For *Ae. aegypti* and *Ae. albopictus*, as well as other mosquitoes, mortality has been shown to vary with temperature in numerous studies. Meta-analysis and pooled analysis [6,7] have shown high variation between studies, suggesting that factors other than temperature influence mortality. These studies show variation in the relationship between mortality and temperature. While there is often a range of temperatures optimal for mosquito survival (lower mortality), the specific temperatures and the width of the optimal range vary between the data sets and analyses performed [6,7,8]. Many factors likely contribute to this variation (e.g., environment, genetics, experimental details, analysis) but the specific relationships are not known.

Chikungunya virus (CHIKV) is a mosquito-borne arbovirus, most commonly transmitted by *Ae. aegypti.* It can also be transmitted by *Ae. albopictus*, and some lineages have evolved to better use *Ae. albopictus* as a vector [9]. While these two species are similar in their ecology, there are differences, notably in host use, with *Ae. aegypti* having a stronger preference for humans, but also in habitat preference or tolerance [10,11,12,13] and in the mortality–temperature relationship [3,6,7]. The two species can coexist, and in those cases both species can contribute to CHIKV transmission. In Florida, the two species can be found in the same containers and overlapping habitat [14] but can also show an abundance gradient from coastal (more *Ae. aegypti*) to inland (more *Ae. albopictus*) areas [15]. The coastal–inland gradient generally overlaps with the urban–rural gradient, as the coast is more urban. Further investigation [16] showed that the factors explaining the presence of each species overlapped at the site level but showed differences in remotely sensed variables. Lwande et al. [17] reviewed the global range and characteristics of the two species, demonstrating that their ranges overlap substantially and there are areas of sympatry. In endemic regions, there are CHIKV cases each year and periodic outbreaks, and the virus can be introduced by travelers to new areas, resulting in outbreaks [18,19]. The relative roles of the two mosquito species varies in space and time. In the US, there was an outbreak in 2014–2016 involving multiple states and territories (e.g., [20,21], including Florida [20]. There are introductions each year from returning travelers [18], presenting an ongoing risk of introduction and epidemics. Improving our understanding of the dynamics of this virus and the factors influencing the likelihood of outbreaks following an introduction is important.

Previously, we developed an arbovirus–mosquito transmission model which has been adapted for different viruses (St. Louis Encephalitis virus/West Nile virus [22,23]; CHIKV [3]; Dengue virus [4]). The CHIKV model included the annual temperature cycle as an input variable and focused on Florida, where the amplitude and mean of the annual cycle are correlated [3]. Using this model, we showed that differences between the two mosquito species and the mean annual temperature affected transmission dynamics, and that aspects of the mortality–temperature relationship altered the importance of other factors.

Our CHIKV model uses a temperature–mortality relationship based on the studies above for both mosquito species. This relationship includes an optimal region where mortality is at its lowest value, increasing to high levels outside the optimal range. In the previous analysis [3] the width of the optimal range was set fairly narrow (2 °C) and only three values of the central point of the optimal survival range were used. This minimal variation in the optimal region, however, showed a strong impact on model behavior and the relative importance of the other parameters in the model. Therefore, we conducted a second analysis of the model, focusing on the mortality and temperature parameters, to assess how these parameters would affect the likelihood and size of an epidemic when varied over a larger parameter space.

## 2. Materials and Methods

The *Aedes*-CHIKV model reported in Lord et al. [3] was used to follow up on results from that study. Specifically, this analysis focuses on the mortality parameters and interactions with temperature and seasonality. As in the previous model, Latin Hypercube sampling (e.g., [3,4,22,23,24]) was used to develop parameter sets. Parameters, descriptions, ranges and distributions for sampling are given in Table 1; the full parameter set used to run the simulations is given in Table S1. Here, most parameters were fixed at values permissive for epidemics based on the previous analysis, and 10 parameters related to mortality and seasonality were varied. For each parameter varied, a distribution is assigned along with minimum, maximum and most probable value (for triangular distributions). Distributions are sampled on the probability axis, generating more values around the most probable values for triangular distributions, then randomly resorted into parameter sets to run the model. Here, we used 250 unique and independent parameter sets.

**Figure 1:**
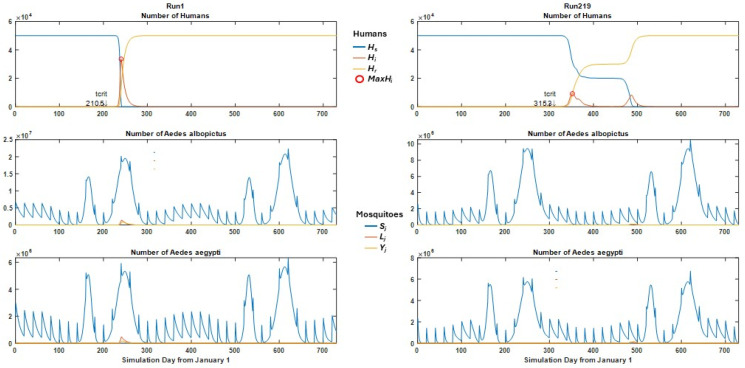
Simulation output. Two example runs (**left panels**: #1; **right panels** #219) shown illustrating two model behaviors, single outbreaks and bimodal outbreaks. Parameters for each run given in Table S1. Top panels: *H_s_*, *H_i_*, *H_r_*: susceptible, infectious and recovered humans, respectively. Middle (*Ae. albopictus*) and bottom panels (*Ae. aegypti*): *Sj*: susceptible mosquitoes; *Lj*: latently infected mosquitoes; *Yj*, infectious mosquitoes. The red circle indicates the maximum infected host population, *MaxH_i_*, and the small arrow indicates the day the virus was introduced, *t_crit_*.

**Figure 2:**
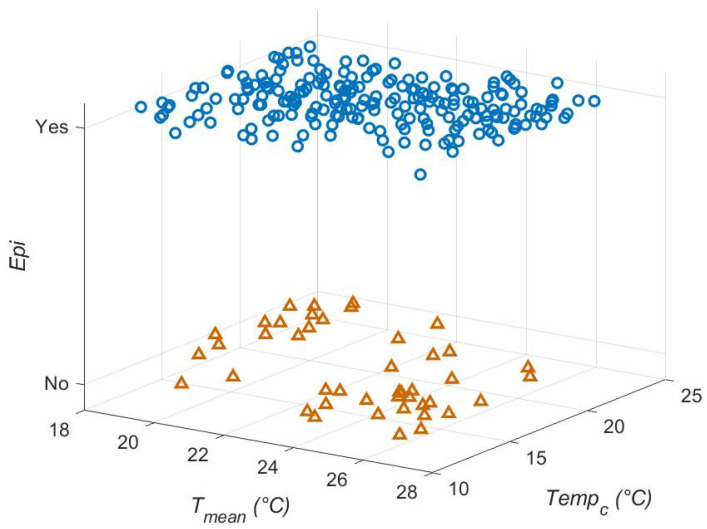
Effect of *Temp_c_*, *T_mean_* and their interaction on *epi*, the likelihood of epidemics. *Epi* is binomial; yes = epidemic occurred (blue circles) and no = epidemic did not occur (brown triangles). Runs in a region around *T_mean_* = 22 °C tended towards epidemic, with some influence of *Temp_c_*.

Full details of the model, mosquito population dynamics, temperature curves and mortality function structure, including parameter estimation, can be found in Lord et al. [3] (see Figures 1 and 2 therein). A brief outline of the model structure and key details is provided here for reference. This is a compartmental, deterministic model with an SIR structure for hosts (humans) and an SEI structure for vectors (two species of mosquito, *Ae. albopictus* and *Ae. aegypti*). An exposed class was not included for humans, as our focus was how different vector species influence transmission cycles and there is no evidence that the vector species affects the duration of the incubation period in humans. Additionally, the incubation period is shorter in CHIKV than in other arboviruses [25], so it would have less impact on the dynamics. A daily temperature is used as a driver for temperature-based virus development and mortality in mosquitoes. Daily temperatures are calculated based on the mean of the annual temperature curve, *T_mean_*, using a relationship derived from Florida temperature data. In Florida, the amplitude of the annual temperature curve is correlated with the mean, and the mean and amplitude are used in a sinusoid function to calculate daily temperatures. Virus is introduced into the system with one infectious human on day *t_crit_*, varied over the entire year in both analyses.

Immature mosquitoes are not explicitly modeled, and adult patterns are functions of mosquito recruitment and mortality. Recruitment is based on general rainfall patterns for Florida and are constructed as a baseline level of recruitment (pulses at *iv* intervals year-round) and two seasonal peaks (spring—early center; mid-late summer) centered on days *q*_1_ and *q*_2_. Model structure allows for variation in the timing and relative size of these recruitment phases, but recruitment structure was not varied in the previous or current analysis; variation in recruitment is planned for future work. The recruitment pattern used here is based on observed rainfall patterns and mosquito abundance in south Florida. Total recruitment (*R_tot_*) is varied separately for the two species, to allow for abundance to impact transmission. Following Lord et al. [3], the biting rate *a_j_* is the inverse of the days between blood meals on humans, *α_j_*. *Aedes albopictus* can be more opportunistic than *Ae. aegypti*, more readily feeding on non-human hosts (e.g., [26,27,28]. *Aedes aegypti*, however, may feed more frequently [29,30]. In both cases, high variation between populations has been observed. To reflect these differences, previously the most probable value of *α_j_* was set lower for *Ae. aegypti* (3 vs. 5, Table 1) and the parameters varied in the analysis. Here, we used these most probable values as fixed parameter values.

Mosquito mortality is temperature dependent, with a central temperature region of optimal survival (low mortality), high mortality at low and high temperatures and linear phases between these regions (see Figure 2 in [3]). The center point of the optimal survival region (*Temp_c_*) and the width to either side (*W*) specify the location and size of the optimal survival region and are the same for the two species. The minimum mortality in the central region and the slope of the increase outside this region (*μ_min_*, *μ_sl_*) further determine the mortality rate at each temperature; these parameters are sampled separately for each species, although the ranges are set the same in this analysis. Previously, three values were used for *Temp_c_* and *W* was fixed at 2 days; here *Temp_c_* was varied between 10 and 20 °C and *W* from 2 to 5 days, to generate a wide range of possible patterns reflecting the variation observed in studies of mortality. This generated mortality–temperature patterns that likely extend beyond the current range of these species, to consider more extreme conditions and explore possible consequences on transmission. Transmission parameters (Table 1 and Table S1) were fixed at permissive values from the previous analysis, to allow for sufficient epidemics in the model set for analysis. There is high variance in mosquito competence for CHIKV, between species, CHIKV strains and with CHIKV mutations that increase the competence of *Ae. albopictus* (e.g., [31,32,33]). Lord et al. [3] used broad ranges for the two transmission parameters, with the ranges and most probable value set the same but the distribution sampled independently. These parameters did influence outcome variables, although *Ae. aegypti* parameters were more influential overall. Here, these parameters were fixed at values that were previously permissive for outbreaks, to focus attention on the mortality parameters while generating enough epidemics for analysis.

Virus development in the mosquito is temperature dependent, and this combined with seasonal reproduction and temperature-dependent mosquito mortality creates seasonality in transmission between mosquitoes and humans.

The model was updated and simulations run in Matlab (version 2019a); analysis was performed in Matlab (version 2021a). The model code for the ODE model is given in the Supplementary Data for Lord et al. [3]. The parameter sets are given in Table S1.

Output variables and statistical analysis: Output variables were chosen to focus on the likelihood of epidemics and the size of epidemics, as in the previous analysis. For human infections, the peak number of infected humans during the simulation (*MaxH_i_*) was assessed for each simulation run. If *MaxH_i_* > 2 the run was scored as epidemic and the variable *epi* set to 1; if not, *epi* = 0. This characterized each run as epidemic (*epi* = 1) or not (*epi* = 0), dividing the 250 simulation runs into the two groups. *Epi* and *MaxH_i_* (*epi* = 1 (epidemic) runs only) were analyzed for their relationship with input parameters.

Using the Latin Hypercube sampling structure, the parameters are sampled independently and should be orthogonal, allowing for regression analysis on the output variables. In some cases, however, parameters for *Ae. aegypti* and *Ae. albopictus* were sampled independently but from the same distributions (Table 1). This can occasionally create correlations between the two realized sets of parameter values. Correlation analysis between the 10 parameter sets showed one strong correlation between two parameters sampled from the same distributions, *R_tot_*_,*alb*_ and *R_tot_*_,*aeg*_ (Table S2). Initially, the analyses were performed with all 10 sampled parameters; however, the matrices for these analyses were ill-conditioned and the results are not reliable. Based on the correlation analysis, we tested dropping one of the correlated parameters from the analysis (*R_tot_*_,*aeg*_); these analyses were better-conditioned and reliable and are reported here.

Logistic regression was used for *epi* (Matlab function fitglm with distribution “binomial), while linear regression was used for *maxHi* (Matlab function fitglm with distribution “normal”). All varied input parameters were included in the regression models, except for dropping the correlated parameter (*R_tot_*_,*aeg*_). In the model situation, all the parameters will have some effect, unlike a field situation where it may be unknown if a measured variable actually influences the outcome variable. Because of this, rather than focusing on *p*-values that meet predetermined thresholds (e.g., α = 0.05 is significant), we use the *p*-values to rank parameters in order of importance. Parameters and associated *p*-values were sorted from smallest to largest *p*-value, then the parameters with the smallest *p*-values were characterized as the most influential on the output variable. For both output variables, we first did a main effects model, to assess which parameters had the most effect on outcomes directly. We then included two-factor interactions in the regression models to assess the effect on the variance explained and the influence of each input parameter. For the main effects model (nine main effects + intercept) we focus on the three parameters with the lowest *p* value; for interaction models we expanded this to the top five.

## 3. Results

### 3.1. Model Behavior

As in the previous sensitivity analysis of this model, we observed a range of model behaviors (Table S3). Many of the simulations showed epidemic behavior (206/250 simulations), in part due to the permissive parameters used. The most common behavior (number of runs, n = 192) was a single epidemic including most hosts (Figure 1, left panels), with some variation in the time between virus introduction and the peak of the epidemic and in the length of the tail. A few simulations (n = 6) were epidemic (*maxHi* > 2) but *maxHi* was very low, indicating the virus died out quickly. Some simulations (n = 14) showed bimodal outbreaks with low virus activity in between (Figure 1, right panels), and the relative sizes of the two outbreaks varied. Frequently, all hosts became infected during the simulation.

### 3.2. Likelihood of Epidemics

In the main effects model, the central temperature (*Temp_c_*) was the most influential variable in the likelihood of an epidemic (*epi*) (Table 2, Figure 2), followed by the slope of the mortality function in *Ae. aegypti* (*μ_sl_*_,*aeg*_, Table 2). The day of virus introduction was the third parameter in the top three. However, the main effects model only explained 8% of the variance (adjusted R^2^ (adj. R^2^) = 0.08, Table 2). The full two-way interactions and main effects model explained 45% of the variation. In this model, the parameter/interactions that were most influential (top five, due to the number of pairwise interactions) included *T_mean_*, *Temp_c_*, *W*, *t_crit_* and the slope of the mortality function for *Ae. albopictus* *(μ_sl_*_,*alb*_) (Table 3 and Table S4A). The interaction with the lowest *p*-value was *Temp_c_* * *T_mean_* (Table 3, Figure 2), although the effect was not strong.

### 3.3. Size of Epidemics

**Figure 3:**
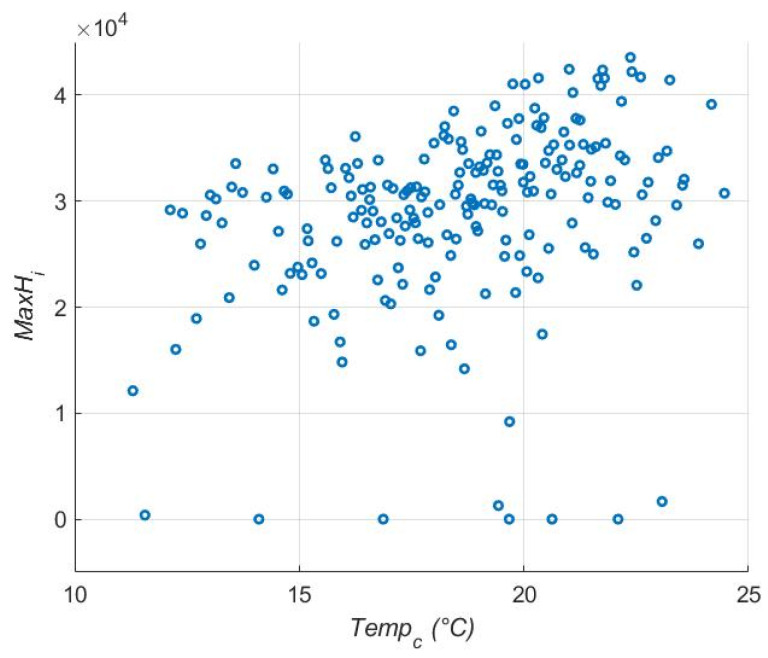
Effect of *Temp_c_*, on *MaxH_i_*, the peak number of hosts infected. Higher *Temp_c_* increases *MaxH_i_*, although the effect is small.

**Figure 4:**
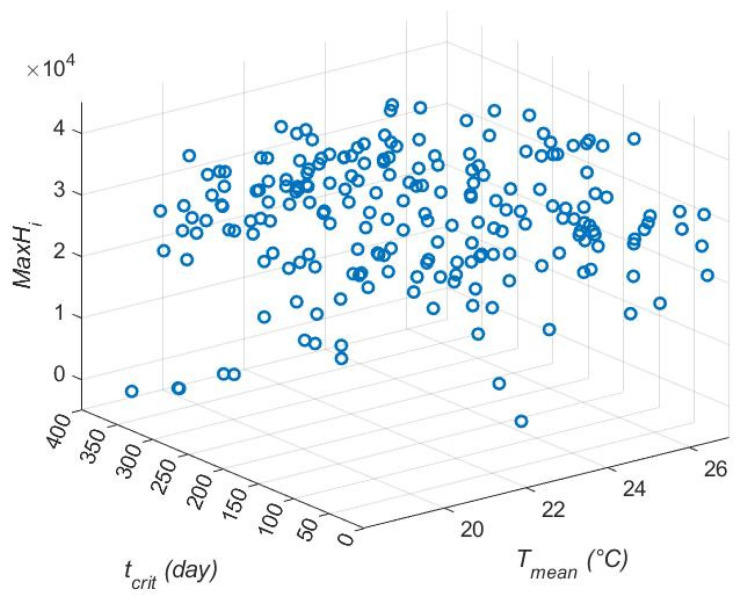
Annual mean temperature (*T_mean_*) and day of introduction (*t_crit_*) effect on *MaxH_i_*. Introductions later in the year with lower mean temperatures generated smaller outbreaks.

For the size of the epidemic (*MaxH_i_*), *Temp_c_* was again the most influential variable in the main effects model (Table 2, Figure 3), followed by the maximum recruitment for *Ae. albopictus* (*ρ_max_*_,*alb*_). The width of the optimal survival region (*W*) was the third parameter in terms of influence on the outcome variable. *Temp_c_* effects are not easily visualized, but there is a trend for increasing *MaxH_i_* with increasing *Temp_c_*. As with *epi*, the effects are not strong and the main effects model only explained 16% of the variation (adj. R^2^ = 0.16). In the interactions model, the most influential variables were interactions between *T_mean_* and *t_crit_* (Figure 4, Table 3 and Table S4B) and *Temp_c_* and *μ_sl_*_,*alb*_. High values of *t_crit_* with low values of *T_mean_* generated a few epidemics with low maximum numbers of hosts infected, although again the signal was not strong. When adjusted for the number of variables, the interaction model explained less of the variance in the data (adj. R^2^ = 0.131) than the main effects model, although the unadjusted R^2^ was higher in the interaction model (R^2^ = 0.32). Multiple interactions in the top five included both *Temp_c_* and *T_mean_*, indicating that these variables are influential in many ways.

## 4. Discussion

There was substantial variation unexplained by the models, indicating that higher order interactions are contributing to the model dynamics. Notably, main effects models explained only minor amounts of variation in the outcome variables. Given the structure of the model, with interacting parameters in most of the differential equations, this is not surprising. For the likelihood of epidemics, the inclusion of two-way interactions indicated that outbreaks appear slightly less likely when *Temp_c_* is low and *T_mean_* is high. There is a region of *T_mean_* around 22 °C where epidemics appear more likely (Figure 2, region with no non-epidemic simulations). Given the temperature structure used, there are likely fewer days with extreme temperatures leading to higher mortality in this *T_mean_* region. The central point of the optimal survival temperature range (*Temp_c_*) consistently ranked high in all models, while other mortality and seasonality parameters and interactions varied between models. However, the variance unexplained by the two-way models was still more than 50%. Higher order interactions may allow for better characterization of the contribution of different parameters, but preliminary testing on this model with a related parameter set (Lord, unpublished data) indicated that a full model with all interactions was required to reach high levels of variance explained. This will require restricting the number of parameters varied and increasing the number of parameter sets, and it is unclear if it will substantially improve our understanding. The importance of interactions, particularly with temperature parameters, was also identified in a coinfection model [34], particularly variation in and interactions with the temperature parameters. This model also used a quadratic relationship between mortality and temperature and considered variations in the temperature function but did not explicitly vary the mortality parameters. By combining variation in the mortality parameters and the temperature function and using a sensitivity analysis that allowed for the exploration of interactions, we highlighted the potential role of temperature–mortality interactions in CHIKV dynamics.

Multiple studies have shown adult mortality in these mosquito species to be temperature dependent (e.g., [6,7,8]), and there was frequently an optimal temperature or range for survival. This optimal temperature region varied in both location and width, both within and between species.

Mosquito populations and temperature dependence in parameters varies between models of CHIKV and other arboviruses. Models have been used to estimate unknown parameters using a compartmental model, subsequently fitting the model to epidemiological data or mosquito abundance data, and then using the fitted models to estimate other parameters and investigate mosquito control (e.g., [35,36,37]). Mosquito mortality can be modeled as temperature dependent (e.g., [3,34,35]) or fixed (e.g., [36,37]). Chen et al. [35] used the same base data as in this model [8] but estimated daily mortality by first fitting a quadradic equation to the data, applying that equation to daily temperatures to estimate a daily temperature-dependent mortality rate and finally fitting a sinusoid function to the estimated daily rates. The model in Chen et al. [35] was used to fit mosquito trap data and infer additional parameters to estimate carrying capacity, so they did not perform the same type of sensitivity analysis as used here. Beyond natural mortality, control strategies can be simulated by increasing mortality at various time points or intervals. This increased mortality decreased the number of cases and shifted the peak time of epidemics in Rio de Janeiro [36]. Using a model to estimate the efficacy of control efforts, then using that efficacy to investigate different scenarios, the timing and number of control events affected the cumulative number of CHIKV cases in France [37].

These models show that the specifics of the mortality function can affect arbovirus dynamics. In the model reported here, this was primarily through interactions between these and other mortality or seasonality parameters, with similar interactions between parameters having effects on coinfection rates [34]. The piecewise mortality–temperature function used here could replicate a wider range of relationships but inherently exhibits a pattern of high mortality at extreme temperatures, a central region of optimal temperatures for survival and linear regions in between. This pattern is also exhibited in other functions such as complex polynomials or quadratic equations, as has been fit in other studies (e.g., [6,7,8,34,35]). However, while a complex polynomial may exhibit a better fit to specific data, the parameters are not easily related to biological concepts and so are difficult to extrapolate. However, consideration of other structures, particularly with non-linear regions, and additional variation between the two species, would be worthwhile. The previous analysis showed that variation between the two species affected transmission dynamics [3]; extended simulation sets and analyses including both types of variation would be valuable in understanding transmission dynamics.

The relationship between the likelihood of epidemics and the seasonal temperature curve was complex, likely non-linear and warrants further research. This should include other relationships between the mean and amplitude of the annual temperature cycle, as the function used here was developed from Florida data only. Understanding this interaction will be important for comparing outcomes from different locations. It is worth noting that this model is deterministic and compartmental and considers only daily temperatures. Agent-based models would be valuable to extend this analysis, to consider finer time scales and individual mosquitoes experiencing varied temperature regimes. However, the trade-off in complexity will limit some aspects of analysis.

## Figures and Tables

**Table 1:** State variables, parameters and parameter distributions used in the model and LHC analysis.

Descriptions	Symbol	Distribution	Range	Center
**State variables**				
Susceptible humans (no.)	*H_s_*			
Infectious humans (no.)	*H_in_*			
Recovered humans (no.)	*H_r_*			
Susceptible mosquitoes, species *j* (no.)	*S_j_*			
Latent mosquitoes, species *j* (no.)	*L_j_*			
Infectious mosquitoes, species *j* (no.)	*Y_j_*			
**Parameters**				
Mean of temperature curve (°C)	*T_mean_*	Uni	18–27	
Day virus is introduced (day)	*t_crit_*	Uni	5–360	
Human recovery rate (day^−1^)	*r_H_*	Fixed		0.125
Total (initial) number of humans (no.)	*H_tot_*	Fixed		50,000
**Both mosquito species**				
Interval between pulses (both species) (days)	*iv*	Fixed		20
Mean day peak one (from January 1) (day)	*q*_1_	Fixed		165
Spread peak one (days)	*σ*_1_	Fixed		7
Mean day peak two (from January 1) (day)	*q*_2_	Fixed		245
Spread peak two (days)	*σ*_2_	Fixed		15
Width of optimal survival range (either side of *Temp_c_*) (°C)	*W*	Uni ^a^	2–5	2
Central point of optimal survival temperature range (°C)	*Temp_c_*	Tri ^b^	10–25	20
***Aedes albopictus***				
Transmission mosquito to human host (day^−1^)	*b_alb_*	Fixed		0.5
Transmission human host to mosquito (day^−1^)	*β_alb_*	Fixed		0.5
Days between blood feeding on humans (day)	*α_alb_*	Fixed		5
Proportion of population in peak one (prop)	*p*_*δ*1,*alb*_	Fixed		0.2
Proportion of population in pulses all year (prop)	*p_base_*_,*alb*_	Fixed		0.15
Minimum mortality (at *Temp_c_* ± *W*) (day^−1^)	*μ_min_*_,*alb*_	Tri	0.04–0.1	0.06
Temperature–mortality slope (mortality/Temp)	*μ_sl_*_,*alb*_	Uni	0.05–0.15	
Virus development at 22.5 °C (day^−1^)	*γ*_22,*alb*_	Fixed		0.25
Temperature–virus development slope (devel. × Temp^−1^)	*γ*_*sl*,*alb*_	Fixed		0.015
Total Albo recruitment through year (no.)	*R_tot_*_,*alb*_	Tri	100–20,000	5000
***Aedes aegypti***				
Transmission mosquito to human host (day^−1^)	*b_aeg_*	Fixed		0.5
Transmission human host to mosquito (day^−1^)	*β_aeg_*	Fixed		0.5
Days between blood feeding on humans (day)	*α_aeg_*	Fixed		3
Proportion of population in peak one (prop)	*p*_1__,*aeg*_	Fixed		0.25
Proportion of population in pulses all year (prop)	*p_base_*_,*aeg*_	Fixed		0.13
Minimum mortality (at *Temp_c_* ± *W*) (day^−1^)	*μ_min_*_,*aeg*_	Tri	0.04–0.1	0.08
Slope of temperature–mortality line (mortality/Temp)	*μ_sl_*_,*aeg*_	Uni	0.05–0.15	
Virus development at 22.5 °C (day^−1^)	*γ*_22,*aeg*_	Fixed		0.25
Temperature–virus development slope (devel. × Temp^−1^)	*γ*_*sl*,*aeg*_	Fixed		0.015
Total *Ae. aegypti* recruitment through year (no.)	*R_tot_*_,*aeg*_	Tri	100–20,000	5000

^a^ Uni = uniform distribution used; parameters are the low–high values (range). ^b^ Tri = triangular distribution used; parameters are the low–high values (range) and the most probable (center) value.

**Table 2:** Main effects models (nine parameters) for *epi* and *MaxH_i_*. Top three parameters for each model indicated by bolded *p*-value.

**Main Effects Model for *MaxH_i_***		
**Descriptions**	**Symbol**	***p* Values**
Central point of optimal survival temperature range	*Temp_c_*	**0.000006**
Total recruitment through year (*Ae. albpopictus*)	*R_tot_*_,*alb*_	**0.000553**
Width of optimal survival range (either side of *Temp_c_*)	*W*	**0.005202**
Slope of temperature–mortality line (*Ae. aegypti*)	*μ_sl_*_,*aeg*_	0.036429
Mean of temperature curve	*T_mean_*	0.250764
Temperature–mortality slope	*μ_sl_*_,*alb*_	0.402013
Day virus is introduced	*t_crit_*	0.543823
Minimum mortality (at *Temp_c_* ± *W*) (*Ae. albopictus*)	*μ_min_*_,*alb*_	0.690523
Minimum mortality (at *Temp_c_* ± *W*) (*Ae. aegypti*)	*μ_min_*_,*aeg*_	0.704036
**Main Effects Model for *epi***		
**Descriptions**	**Symbol**	***p* Values**
Central point of optimal survival temperature range	*Temp_c_*	**0.00692**
Slope of temperature–mortality line	*μ_sl_*_,*aeg*_	**0.010205**
Day virus is introduced	*t_crit_*	**0.032391**
Mean of temperature curve	*T_mean_*	0.679624
Minimum mortality (at *Temp_c_* ± *W*) (*Ae. aegypti*)	*μ_min_*_,*aeg*_	0.786395
Minimum mortality (at *Temp_c_* ± *W*) (*Ae. albopictus*)	*μ_min_*_,*alb*_	0.821233
Temperature-mortality slope (*Ae. albopictus*)	*μ_sl_*_,*alb*_	0.852199
Total recruitment through year (*Ae. albopictus*)	*R_tot_*_,*alb*_	0.868015

**Table 3:** Top five parameters (highest *p* value) for main effects and interaction models for *epi* and *MaxH_i_*.

**Main Effects and Two-Way Interaction Model for *MaxH_i_***				
**Descriptions**	**Predictor 1**	**Predictor 2**	***p* Values**	**Main Effect/Two-Way** **Interactions**
Mean of temperature curve Day virus is introduced	*T_mean_*	*t_crit_*	0.008151	Two-way interaction
Temperature–mortality slope (*Ae. albopictus*) Center point of optimal temperature range	*μ_sl_*_,*alb*_	*Temp_c_*	0.052552	Two-way interaction
Mean of temperature curve	*T_mean_*	*Temp_c_*	0.053523	Two-way interaction
Center point of optimal temperature range Center point of optimal temperature range	*Temp_c_*		0.157706	Main effect
Mean of temperature curve Temperature-mortality slope (*Ae. albopictus*)	*T_mean_*	*μ_sl_*_,*alb*_	0.226489	Two-way interaction
**Main Effects and Two-Way Interaction Model for *epi***				
**Descriptions**	**Predictor 1**	**Predictor 2**	***p* Values**	**Main Effects/Two-Way** **Interactions**
Mean of temperature curve Center temperature for mortality	*T_mean_*	*Temp_c_*	0.000001	Two-way interaction
Center temperature for mortality Width of optimal survival range (either side of *Temp_c_*)	*Temp_c_*	*W*	0.000112	Two-way interaction
Center temperature for mortality	*Temp_c_*		0.000141	Main effect
Day virus is introduced	*t_crit_*		0.011305	Main effect
Mean of temperature curve Temperature–mortality slope (*Ae. albopictus*)	*T_mean_*	*μ_sl_*_,*alb*_	0.01481	Two-way interaction

## Data Availability

All data used in analysis are provided in the Supplementary Materials. Model code is available in the Supplementary Materials for Lord et al. [3].

## Supplementary Materials

The following supporting information can be downloaded at https://www.mdpi.com/article/10.3390/v17111486/s1: Table S1. full parameter sets used to run the simulations; Table S2. correlation coefficients between parameters; Table S3. model output used in analysis; Table S4. results from two-way interaction models.

## Abbreviations

The following abbreviations are used in this manuscript:

CHIKV	Chikungunya virus
Adj. R^2^	Adjusted R^2^
